
JOURNAL INFORMATION
==============================
NLM Title Abbreviation: J Diabetes Investig
Journal ID: J Diabetes Investig
NLM Journal ID: 101520702
Journal ID: JDI

Journal of Diabetes Investigation

ISSN: 2040-1116
EISSN: 2040-1124
Publisher: Wiley

ARTICLE INFORMATION
==============================
PMCID: PMC11474885
DOI: 10.1111/jdi.14257
Article ID: JDI14257
Article version: 1
Subjects: Issue Information, Issue Information

Issue Information

Electronic publication date: 2024 Jul 1
Publication date: 2024 Jul
Volume: 15
Issue: 7
First page: 793
Last page: 794
Copyright: © 2024 The Authors. Journal of Diabetes Investigation published by Asian Association for the Study of Diabetes (AASD) and John Wiley & Sons Australia, Ltd
License: This is an open access article under the terms of the http://creativecommons.org/licenses/by/4.0/ License, which permits use, distribution and reproduction in any medium, provided the original work is properly cited.
License URL: https://creativecommons.org/licenses/by/4.0/

Figure count: 0
Table count: 0
Page count: 5

source-schema-version-number: 2.0
cover-date: July 2024
details-of-publishers-convertor: Converter:WILEY_ML3GV2_TO_JATSPMC version:6.4.9 mode:remove_FC converted:15.10.2024

==============================

### JDI

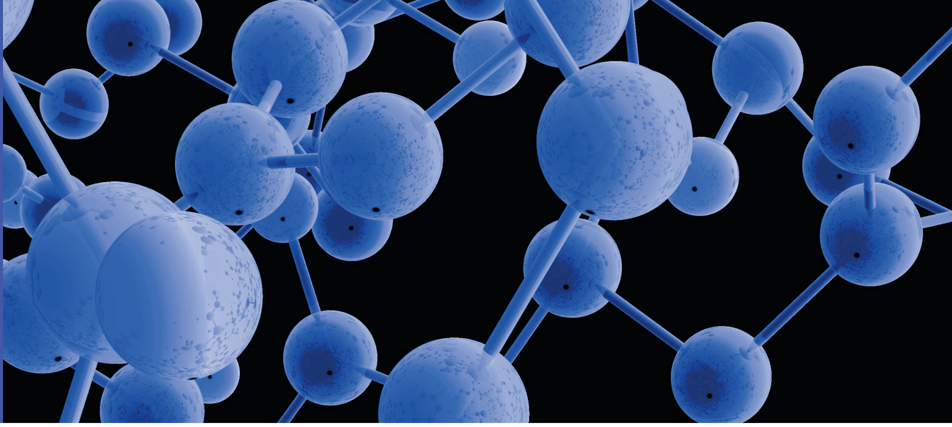

Volume 15 | Issue 7
July 2024

**Journal of Diabetes Investigation**
Official Journal of the Asian Association for the Study of Diabetes

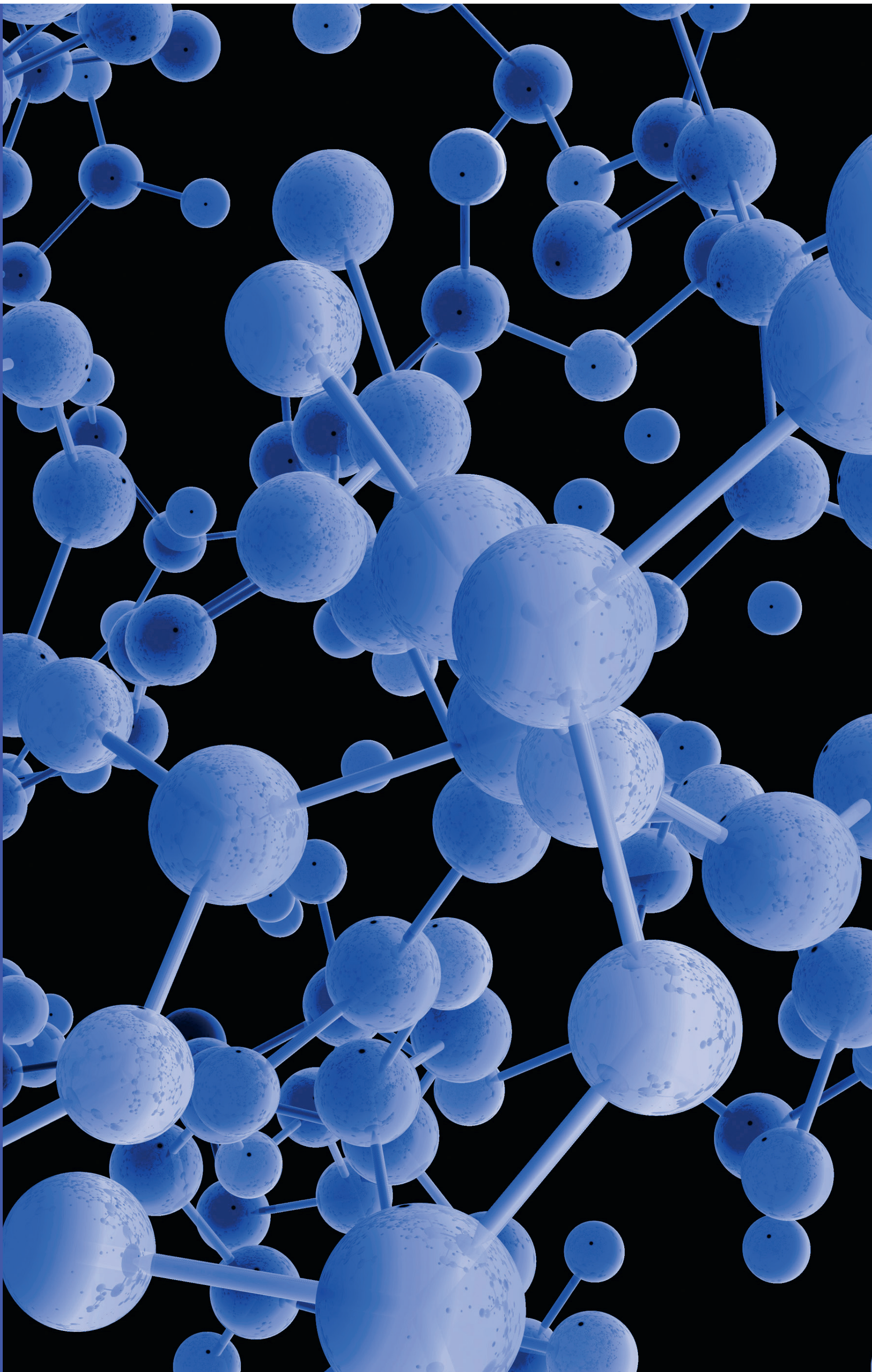

Cognition in elderly type 1 diabetes

THE ASIAN ASSOCIATION FOR THE STUDY OF DIABETES
WILEY

**JDI Journal of Diabetes Investigation** Open access
Official Journal of the Asian Association for the Study of Diabetes

**Editor-in-Chief**
Nigishi Hotta, *Japan Organization of Occupational Health and Safety, Chubu Rosai Hospital, Nagoya, Japan*

**Co-Editor**
Domenico Accili, *Columbia University, New York, USA*

**Expert Editor**
Bo Ahren, *Lund, Sweden*
Young Min Cho, *Seoul, Korea*
Sean Dinneen, *Galway, Ireland*
Marc Gilbert, *Montpelier, France*
Limei Liu, *Shanghai, China*
Ronald Ma, *Hong Kong, China*
Louis Philipson, *Chicago, USA*
Wayne H-H Sheu, *Taichung, Taiwan*
Bernard Thorens, *Lausanne, Switzerland*
Daisuke Yabe, *Kyoto, Japan*

**Editorial Board**
Eiichi Araki, *Kumamoto, Japan*
Xiaoping Chen, *Beijing, China*
Lee-Ming Chuang, *Taipei, Taiwan*
Masakazu Haneda, *Asahikawa, Japan*
Larry Low-Tone Ho, *Taipei, Taiwan*
Tianpei Hong, *Beijing, China*
Akihisa Imagawa, *Osaka, Japan*
Nobuya Inagaki, *Kyoto, Japan*
Linong Ji, *Beijing, China*
Weiping Jia, *Shanghai, China*
Yi-Der Jiang, *Taipei, Taiwan*
Takashi Kadowaki, *Tokyo, Japan*
Kohei Kaku, *Kurashiki, Japan*
Keizo Kanasaki, *Izumo, Japan*
Doo-Man Kim, *Seoul, Korea*
Karen Lam, *Hong Kong, China*
Hong Kyu Lee, *Gyeonggi-do, Korea*
In-Kyu Lee, *Daegu, Korea*
Moon-Kyu Lee, *Seoul, Korea*
Jiro Nakamura, *Nagakute, Japan*
Kishio Nanjo, *Wakayama, Japan*
Wataru Ogawa, *Kobe, Japan*
Yutaka Seino, *Osaka, Japan*
Shyi-Jang Shin, *Kaohsiung, Taiwan*
Hirohito Sone, *Niigata, Japan*
Kathryn Tan, *Hong Kong, China*
Yukio Tanizawa, *Ube, Japan*
Kohjiro Ueki, *Tokyo, Japan*
Jun Wada, *Okayama, Japan*
Hirotaka Watada, *Tokyo, Japan*
Jianping Weng, *Guangzhou, China*
Kyu-Chang Won, *Daegu, Korea*
Soroku Yagihashi, *Hirosaki, Japan*
Hiroshi Yamamoto, *Kanazawa, Japan*
Toshimasa Yamauchi, *Tokyo, Japan*
Wenying Yang, *Beijing, China*
Kazuki Yasuda, *Tokyo, Japan*
Koutaro Yokote, *Chiba, Japan*
Kun-Ho Yoon, *Seoul, Korea*
Dalong Zhu, *Nanjing, China*

**International Editorial Board**
Michael A. Brownlee, *New York, USA*
Subrata Chakrabarti, *Ontario, Canada*
Mark E. Cooper, *Prahran, Australia*
Philippe Froguel, *London, UK*
Wilfred Fujimoto, *Hawaii, USA*
Michael S. German, *San Francisco, USA*
Andrew Hattersley, *Exeter, UK*
Jean-Claude Henquin, *Brussels, Belgium*
Jens Juul Holst, *Copenhagen, Denmark*
Frank B. Hu, *Boston, USA*
C. Ronald Kahn, *Boston, USA*
George L. King, *Boston, USA*
Rayaz A. Malik, *Manchester, UK*
Michael Mauer, *Minneapolis, USA*
Carl Erik Mogensen, *Aarhus, Denmark*
Steven E. Shoelson, *Boston, USA*
Jay S. Skyler, *Miami, USA*
Paul Zimmet, *Melbourne, Australia*

**Assistant Editorial Board**
Tien-Jyun Chang, *Taipei, Taiwan*
Yoshiyuki Hamamoto, *Osaka, Japan*
Norio Harada, *Fukui, Japan*
Yi-Jen Hung, *Taipei, Taiwan*
Chi-Min Hwu, *Taipei, Taiwan*
Hideki Kamiya, *Aichi, Japan*
Dae Jung Kim, *Suwon, Korea*
Seung-Hwan Lee, *Seoul, Korea*
Hung-Yuan Li, *Taipei, Taiwan*
Takashi Matsuzaka, *Tsukuba, Japan*
Keiko Naruse, *Aichi, Japan*
Takeshi Nishikawa, *Kumamoto, Japan*
Eung-Jung Rhee, *Seoul, Korea*
Yusuke Seino, *Aichi, Japan*
Hironori Waki, *Tokyo, Japan*

**Aims and Scope:** *Journal of Diabetes Investigation* is your core diabetes journal from Asia; the official journal of the Asian Association for the Study of Diabetes (AASD). The journal publishes original research, country reports, commentaries, reviews, mini-reviews, case reports, letters, as well as editorials and news. The journal embraces clinical and experimental research in diabetes and related areas. This includes aspects of prevention, treatment, as well as molecular aspects and pathophysiology. Translational research focused on the exchange of ideas between clinicians and researchers is also welcome.

**Cover:** Insulin molecule © Pasieka/Science Photo Library

This journal is available online at Wiley Online Library. Visit wileyonlinelibrary.com to search the articles and register for table of contents and e-mail alerts.

**For submission instructions, and all other information visit wileyonlinelibrary.com/journal/jdi**

JDI.JEB.Jul24

**Open Access and Copyright**

All articles published by *Journal of Diabetes Investigation* are fully open access: immediately freely available to read, download and share. All *Journal of Diabetes Investigation* articles accepted from 24 September 2013 are published under the terms of the Creative Commons Attribution 4.0 Unported (CC BY 4.0) license, which permits use, distribution and reproduction in any medium, provided the original work is properly cited. Articles accepted before this date were published under the agreement as stated in the final article. Copyright on any *Journal of Diabetes Investigation* article accepted after 24 September 2013, the date on which *Journal of Diabetes Investigation* became an open access journal, is retained by the author(s). Authors grant to *Journal of Diabetes Investigation* and Wiley a license to publish the article and for Wiley to identify itself as the original publisher. Authors also grant any third party the right to use the article freely as long as its integrity is maintained and its original authors, citation details and publisher are identified. Further information about open access licenses and copyright can be found at: https://authorservices.wiley.com/author-resources/Journal-Authors/licensing/licensing-info-faqs.html#4. Information on the licenses used by Journal of Diabetes Investigation can be found at: wileyonlinelibrary.com/journal/jdi

**Disclaimer**

The Publisher, the Asian Association for the Study of Diabetes, and Editors cannot be held responsible for any errors in or any consequences arising from the use of information contained in this journal. The views and opinions expressed do not necessarily reflect those of the Publisher, the Asian Association for the Study of Diabetes or Editors, neither does the publication of advertisements constitute any endorsement by the Publisher, the Asian Association for the Study of Diabetes, Editors, or Authors, of the products advertised.

Wiley Open Access articles posted to repositories or websites are without warranty from Wiley of any kind, either express or implied, including, but not limited to, warranties of merchantability, fitness for a particular purpose, or non-infringement. To the fullest extent permitted by law Wiley disclaims all liability for any loss or damage arising out of, or in connection, with the use of or inability to use the content.

JDI.PI.Jul24

VOLUME 15 – ISSUE 7 – JULY 2024

### Journal of Diabetes Investigation

Open access

### Reviews

**Diabetes treatment by conversion of gut epithelial cells to insulin-producing cells**
Domenico Accili, Shivatra C Talchai, Ryotaro Bouchi, April Yun-Kyoung Lee, Wen Du, Takumi Kitamoto, Wendy M McKimpson, Sandro Belvedere, and Hua V Lin 797

**High-density lipoprotein in diabetes: Structural and functional relevance**
David Tak Wai Lui and Kathryn Choon Beng Tan 805

### Commentary

**Gut microbiota dysbiosis as a novel pathogenic factor of diabetic peripheral neuropathy**
Naoko Niimi and Kazunori Sango 817

### Update

**Advances in diagnostic methods for early-stage diabetic polyneuropathy**
Keiko Naruse 820

### Original Articles

### Basic Science and Research

***Polygonatum sibiricum* (Huang Jing) polysaccharide reduces diabetic cardiomyopathy through increasing cyclic guanosine monophosphate-protein kinase G signaling in diabetic mice**
Shengping Lei, Xin Lu, Lei Yan, Tian Liu, Yan Niu, and Jun Yu 823

### Clinical Science and Care

**Prediction of future insulin-deficiency in glutamic acid decarboxylase autoantibody enzyme-linked immunosorbent assay-positive patients with slowly-progressive type 1 diabetes**
Eiji Kawasaki, Takuya Awata, Hiroshi Ikegami, Akihisa Imagawa, Yoichi Oikawa, Haruhiko Osawa, Takeshi Katsuki, Norio Kanatsuna, Ryoichi Kawamura, Junji Kozawa, Noriko Kodani, Tetsuro Kobayashi, Akira Shimada, Masayuki Shimoda, Kazuma Takahashi, Daisuke Chujo, Tetsuro Tsujimoto, Kyoichiro Tsuchiya, Aiko Terakawa, Jungo Terasaki, Kan Nagasawa, Shinsuke Noso, Tomoyasu Fukui, Ichiro Horie, Kazuki Yasuda, Hisafumi Yasuda, Hidekatsu Yanai, Toshiaki Hanafusa, and Hiroshi Kajio, on behalf of the Japanese Type 1 Diabetes Database Study (TIDE-J) 835

**Effect of sodium–glucose cotransporter 2 inhibitors on serum low-density lipoprotein cholesterol in Japanese patients with type 2 diabetes mellitus**
Tasuku Imada, Naoto Katakami, Hirotaka Watanabe, Shuhei Nishina, Shugo Sasaki, Mitsuyoshi Takahara, Iichiro Shimomura, and Tsunehiko Yamamoto 843

**Improvements of myocardial strain and work in diabetes patients with normal ejection fraction after empagliflozin treatment**
Xueqing Cheng, Peina Huang, Hongyun Liu, Xiaojun Bi, Yiping Gao, Ruirui Lu, Yipeng Gao, Yani Liu, and Youbin Deng 851

### Short Report

**Implementation rate of diabetic self-management education and support for Japanese people with diabetes using the National Database**
Noriko Ihana-Sugiyama, Takehiro Sugiyama, Kenjiro Imai, Mitsuru Ohsugi, Kohjiro Ueki, Toshimasa Yamauchi, and Takashi Kadowaki 861

### Original Articles

**Serum vitamin D is substantially reduced and predicts flares in diabetic retinopathy patients**
Yong Zhuang, Zihao Zhuang, Qingyan Cai, Xin Hu, and Huibin Huang 867

**An exploratory study of factors in disordered eating behavior in diabetes self-management in Japan**
Sachiko Tsukamoto-Kawashima, Kaori Ikeda, Masakatsu Sone, Masahito Ogura, Emi Okamura, Aki Kondo, Yoko Ueba, and Nobuya Inagaki 874

**Characterization of the visually impaired patients with diabetes mellitus in Japan**
Yuka Sugihara, Yoshihiro Takamura, Yutaka Yamada, Masakazu Morioka, Makoto Gozawa, Kumiko Kato, Takao Hirano, Fumiko Murao, Miho Shimizu, Sentaro Kusuhara, Tomoya Murakami, Yuki Takenaka, Naoko Okabe, Tatsuya Jujo, Hiroto Terasaki, Daisuke Nagasato, Zhenyu Dong, Shigeo Yoshida, Shuntaro Ogura, Kanako Yasuda, Gaku Ishigooka, Osamu Sawada, Fumiaki Higashijima, and Masaru Inatani **882**

**The impact of handgrip strength and waist circumference on glycemic control: Prospective, observational study using outpatient clinical data in Japanese patients with type 2 diabetes mellitus**
Shuhei Nakanishi, Masashi Shimoda, Tomohiko Kimura, Junpei Sanada, Yoshiro Fushimi, Yuichiro Iwamoto, Hideyuki Iwamoto, Kazunori Dan, Tomoatsu Mune, Kohei Kaku, and Hideaki Kaneto **892**

**Relationship between diabetes treatment status and occupational accidents**
Ayana Ogasawara, Tomohisa Nagata, Kiminori Odagami, Masako Nagata, Nuri Purwito Adi, and Koji Mori **899**

**Association between infant birth weight and gestational weight gain in Japanese women with diabetes mellitus**
Kei Fujikawa Shingu, Masako Waguri, Mitsuyoshi Takahara, Aurélie Piedvache, Naoto Katakami, and Iichiro Shimomura **906**

**Post-load glucose is a stronger predictor of adverse pregnancy outcomes than first-trimester HbA1c in women without gestational diabetes**
Shahin Keshtkar Rajabi, Elham Toghraee, and Golnoosh Nejatipour **914**

**Global cognition and executive functions of older adults with type 1 diabetes mellitus without dementia**
Kaoru Nagasawa, Kimio Matsumura, Takayasu Uchida, Yuya Suzuki, Akihiro Nishimura, Minoru Okubo, Yukifusa Igeta, Tetsuro Kobayashi, Takashi Sakurai, and Yasumichi Mori **922**

**Between blood glucose and mortality in critically ill patients: Retrospective analysis of the MIMIC-IV database**
Yuanyuan Li, Wenqiang Li, and Baocai Xu **931**

**Association between glycated hemoglobin and risk of all-cause mortality in community patients with type 2 diabetes: A prospective cohort study**
Qingfang He, Mingfei Gao, Xiaoyan Zhou, Lixin Wang, Yujia Fang, and Ruying Hu **939**

**Association between gastrointestinal symptoms and insomnia in patients with type 2 diabetes: The KAMOGAWA-DM cohort study**
Megumi Minamida, Hiroshi Okada, Masahide Hamaguchi, Junya Hironaka, Yuriko Kondo, Hanako Nakajima, Takuro Okamura, Takafumi Sennmaru, Naoko Nakanishi, Emi Ushigome, and Michiaki Fukui **946**

**Modification effect of receipt of diabetes care on the association between COVID-19 infection and HbA1c level during the first year of the coronavirus pandemic using a nationwide population-based database**
Akira Okada, Satoko Yamaguchi, Sachiko Ono, Kayo Ikeda Kurakawa, Hideo Yasunaga, Masaomi Nangaku, Toshimasa Yamauchi, and Takashi Kadowaki **953**

### Case Report

**Coexistence of type 2 diabetes mellitus, arginine vasopressin deficiency, and Marfan syndrome: A case report**
Yunyi Le, Jingjing Zhang, Tianpei Hong, and Jin Yang **964**
